# Vertical Open Patella Fracture, Treatment, Rehabilitation and the Moment to Fixation

**DOI:** 10.14740/jocmr2005w

**Published:** 2014-11-19

**Authors:** Joao Alberto Larangeira, Liliane Bellenzier, Vanessa da Silva Rigo, Elias Josue Ramos Neto, Francisco Fritsch Machry Krum, Tiango Aguiar Ribeiro

**Affiliations:** a Servico de Ortopedia e Traumatologia do Hospital Universitario de Santa Maria (SOT - HUSM), Universidade Federal de Santa Maria (UFSM), Santa Maria, Rio Grande do Sul (RS), Brazil; b Departamento de Cirurgia, Centro de Ciencias da Saude (CCS), Universidade Federal de Santa Maria (UFSM), Santa Maria, Rio Grande do Sul (RS), Brazil

**Keywords:** Open fractures, Patella, Intra-articular fractures, Tension band, Primary wound closure

## Abstract

Patella fracture is relatively uncommon and the vertical trace fracture represents almost 12-17%. The open patella fracture expresses 6-30%. The association of these two uncommon conditions was the aim of this case report even as the treatment and the moment of fixation (definitive surgical treatment). A 27-year-old man after a motorcycle accident showed an open patella fracture classified as a Gustilo and Anderson type IIIA lesion. The patient was immediately treated with precocious surgery fixation with a modified tension band which consists of two parallel K-wires positioned orthogonal to the fracture line and a cerclage wire shaped anteriorly at patella as an eight. The premature fixation benefited the infection prevention and provided earlier joint motion, which increased the nutrition of articular cartilage. Six months postoperatively, the patient had a satisfactory joint motion with full extension and 116° of joint flexion and returned to his daily life activities without restriction. Twelve months postoperatively, the patient had full extension and 120° of knee flexion without pain, joint effusion and instability. Muscle strength force was considered normal at grade V. In conclusion, early chirurgic treatment and precocious articular mobilization improve prognosis, suggesting that the employment of these practices should be adopted whenever possible in most of the open fractures.

## Introduction

**Figure 1:**
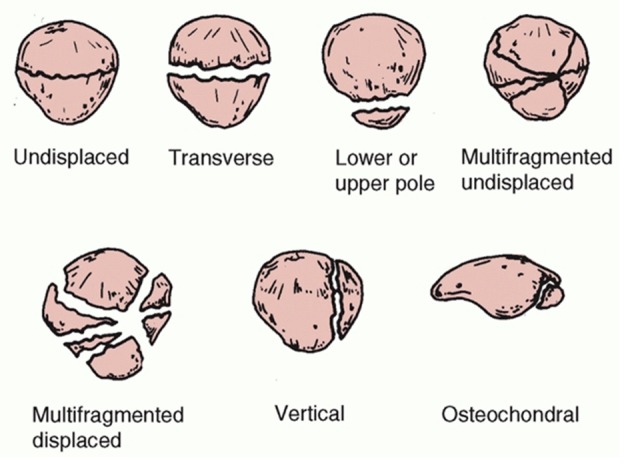
Descriptive classification of patellar fractures [5]. Reproduced with permission.

Patella fracture is relatively uncommon and represents approximately 0.5-1.5% of all bone injuries [1, 2], and men are more affected than women [3, 4]. The classification can be done by characterizing the fracture pattern in osteochondral, multifragmented, stellate, transversal, vertical and polar fracture (Fig. 1). Other classifications may take into account the presence of deviation between the fragments, deviated and not deviated, and the mechanism of injury. The transversal type is more frequent corresponding to 50-80%, comminuted 30-35% and vertical fractures 12-17% [2, 3] and these are rarely displaced [5]. Open patella fracture expresses 6-30% of all patella fractures [6-9].

The aims of this case report were to describe an uncommon condition of associated injuries of patella, an open vertical patella fracture, to revise the literature about patella fracture treatment and to discuss the moment to realize the definitive fixation of an open fracture. The present case has the patient permission to be published through the informed consent.

## Case Report

**Figure 2:**
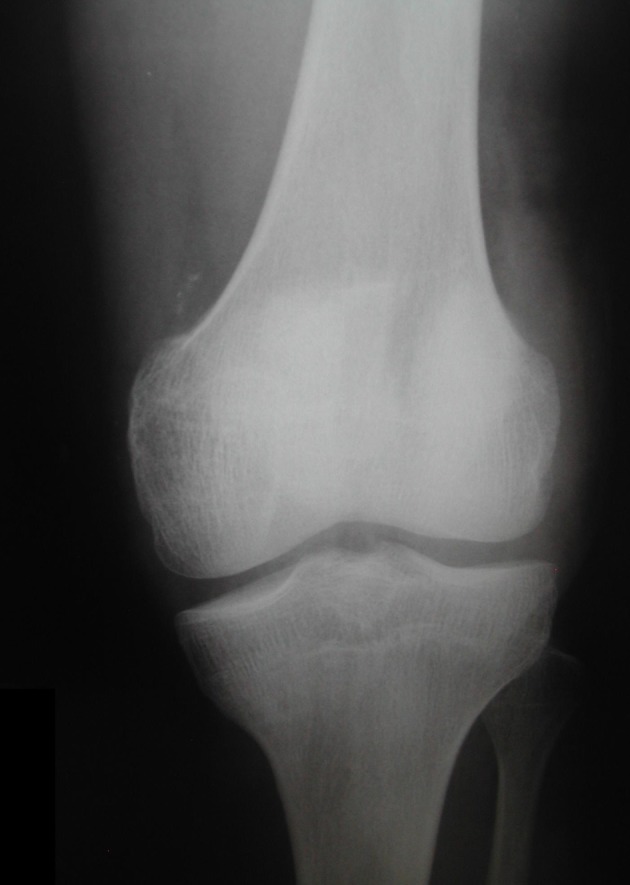
Anteroposterior radiograph demonstrated a vertical displaced patella fracture. Note that the deviated fragments are separated more than 3 mm.

**Figure 3:**
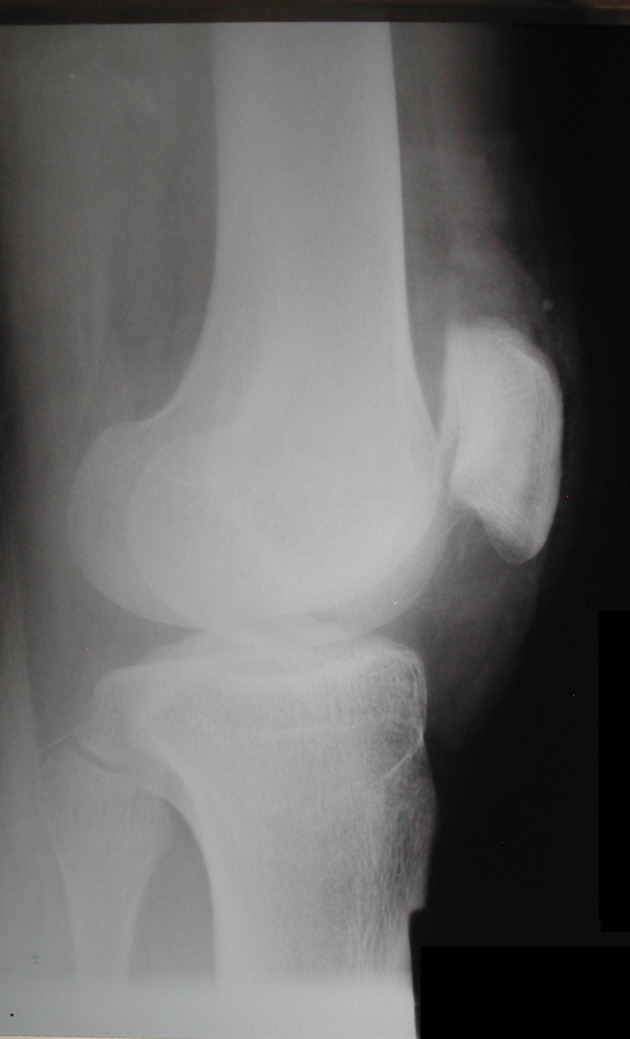
Lateral radiograph demonstrated the fracture by the articular gap in patella. Note the difference between the lateral and the medial side of patella caused by the displaced vertical trace of fracture.

A 27-year-old man, previously healthy, arrived at our hospital after a crash accident between his motorcycle and a car. The patient complained that his right knee was pressed among vehicles and then he fell in the ground. On examination, in the patient’s right knee, the complete limitation in range of motion, swelling and severe pain were identified. A 10-cm injury was observed in the anterior face of the knee with exposition of soft tissues and a fractured patella. The affected member had little traces of smudge ground. Neurovascular lesion was not observed in examination. The complex was initially classified as a lesion type II of Gustilo and Anderson [10]. Superficial abrasions were observed in the left shoulder and in the lips, without clinical importance. The patient was scrutinized radiologically and the knee images demonstrated a complete deviated not comminuted vertical patella fracture (Fig. 2, 3).

After the initial examination, the patient was conducted to cirurgic room. Surgery, anti-tetanus and intravenous antibiotic prophylaxis with cefazolin, gentamicin and metronidazole were then performed. The margins of wound were extended, and then an adequate debridement with the removal of gross contamination and necrotic tissue was made and small pieces of the external paint of vehicles associated with small amount of soil were found in the injury and also were removed. Supplementing the debridement, the irrigation was made with 10 L of 0.9% physiologic solution. There was no bone loss and comminution. The real extension of the damage was evaluated and the lesion classification changed to type IIIA of Gustilo and Anderson [10].

The fracture fixation was performed and the adopted technique was the modified tension band with Kirschner and cerclage wire as described by Cramer and Moed [11]. The joint congruence was respected and visualized by the surgeon digital touch. Primary closure of the wound was performed intraoperatively and a drain was used. A splint was used to assist in analgesia and healing of hurt. Patient’s in-hospital stay was 3 days, the same time of antibiotics use. In the discharged the drain was removed and no sign of infection was present.

**Figure 4:**
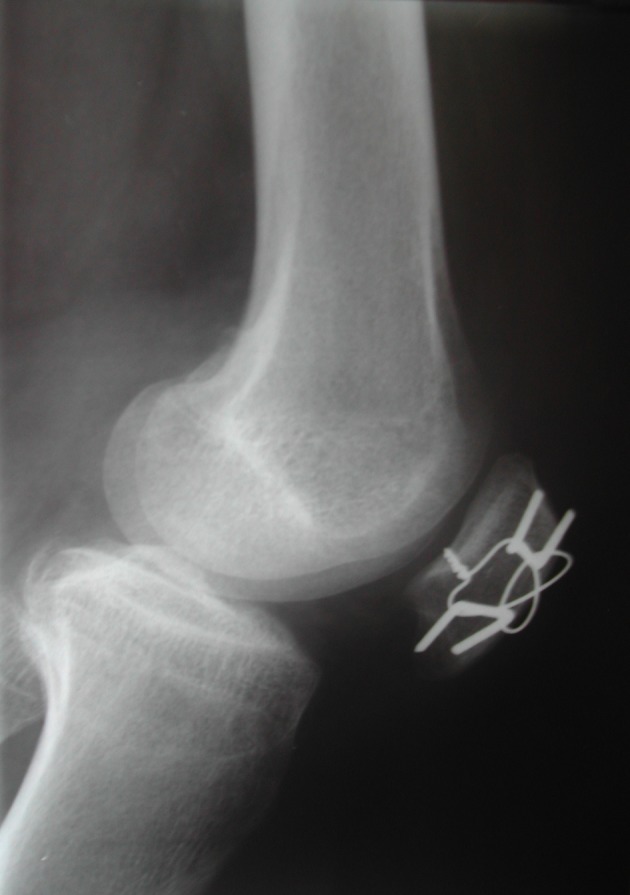
Seven days postoperatively, lateral radiograph demonstrated the total reduction of the patella fracture without joint gap and the use of the modified tension band.

**Figure 5:**
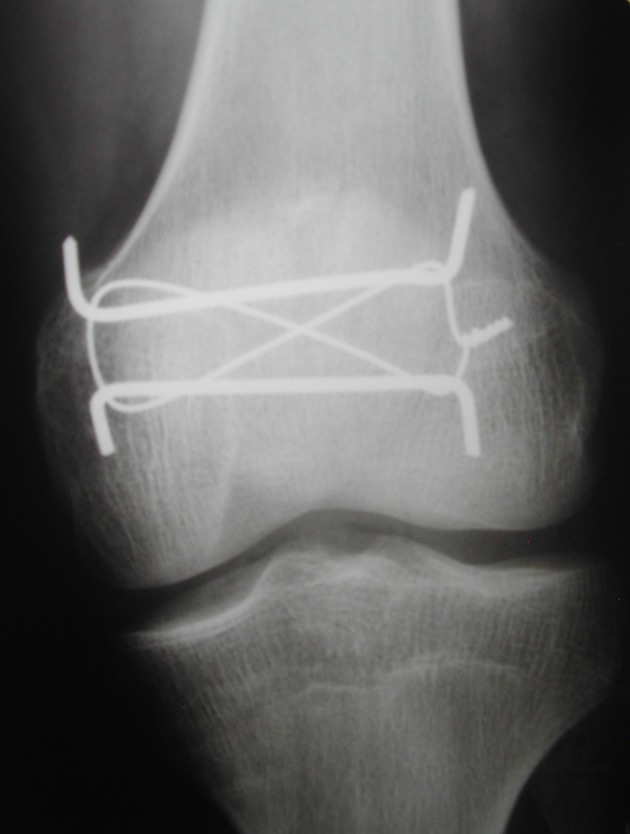
Seven days postoperatively, anteroposterior radiograph demonstrated the reduction of the separated fragments and the 3 mm distance between the fragments was gone. This incidence has shown better modified tension band.

**Figure 6:**
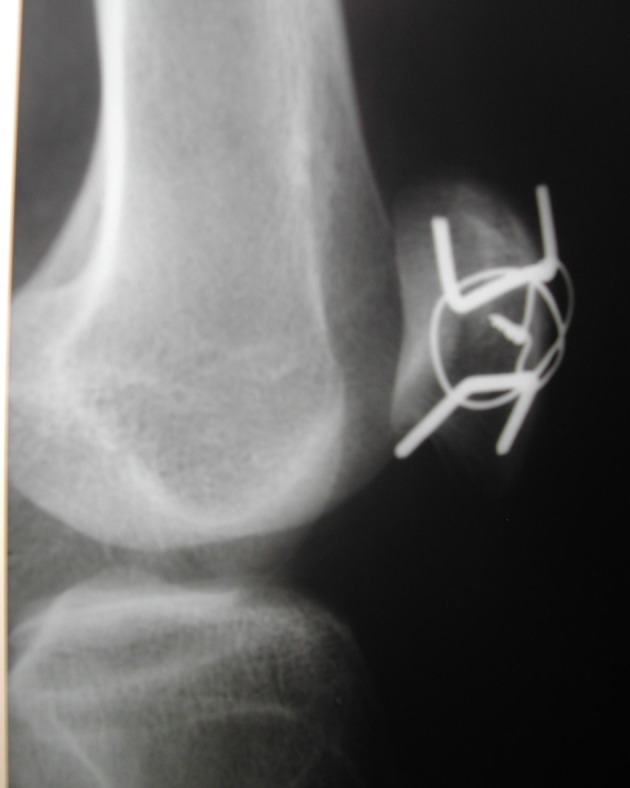
Seven days postoperatively, oblique radiograph demonstrated better articular surface without joint gaps.

**Figure 7:**
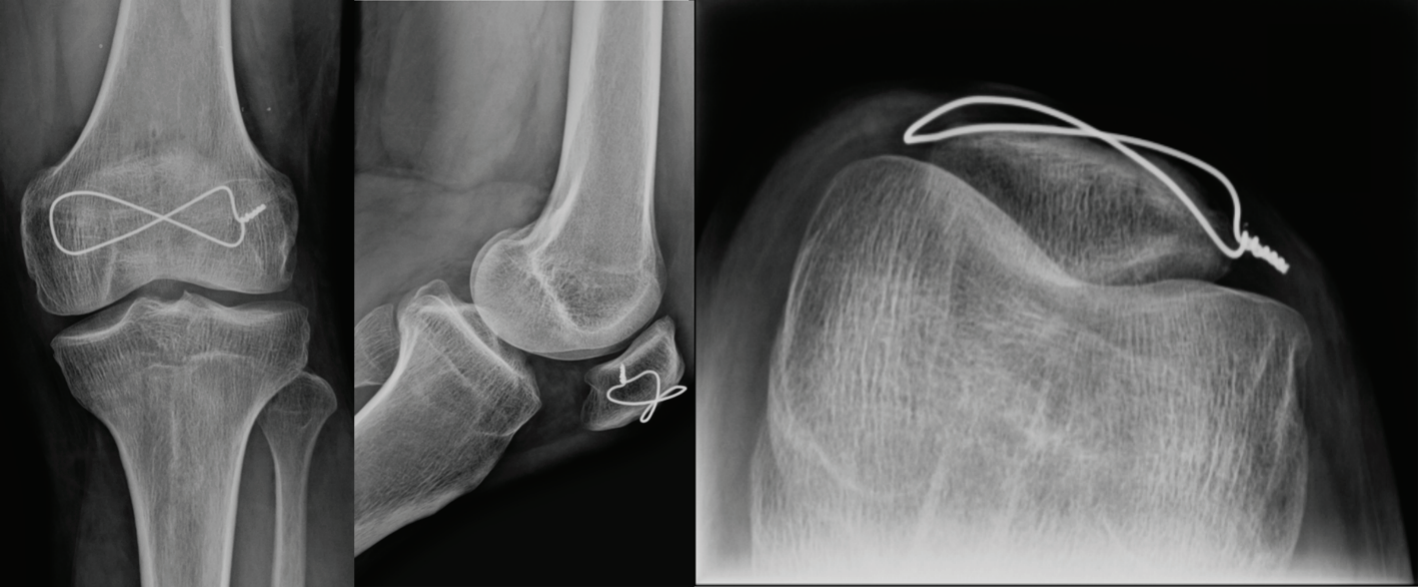
Twelve months postoperatively, no arthritis sign, the complete consolidation without articular gaps and the removal of the K-wires were shown. Anteroposterior radiograph (left side), lateral radiograph (center) and axial radiograph (right side).

Seven days postoperatively, a radiograph was made and the fracture reduction was confirmed and no joint gap was observed (Fig. 4-6). The splint was removed 2 weeks after surgery and the injury has healed without infection, and the full weight bearing and the active knee movement was initiated. Two months after surgery, the radiographs demonstrated the fracture consolidation. At 4 months, the related pain due to the salience of K-wires in the knee skin indicated the removal of the K-wires. Twelve months postoperatively, the patient had full extension and 120° of knee flexion without pain, joint effusion and instability. Muscle strength force was considered normal, grade V [12]. No signs of arthritis were observed in X-ray (Fig. 7). The patient came back to his daily life activities and job without restrictions.

## Discussion

Open patella fracture compared with closed fracture of the same joint is in majority attributed to high-energy trauma such as in car accidents [6, 9] and the direct trauma is the main mechanism [3]. Comminuted patella fracture and associated fractures of the other joints can also be found due to the nature of the trauma that caused the patella fracture [7], unlike this case that did not demonstrate this situation.

Patella fractures can be treated conservatively or surgically [13, 14]. Treatment choice is performed based on the fracture classification and findings on physical examination [5]. The conservative treatment can be chosen if the extensor mechanism is functioning for the following cases: non-displaced fractures, fractures in which the articular gaps are lesser than 2 mm and fractures in which deviated fragments are lesser than 3 mm. When these conditions cannot be satisfied, the surgical treatment should be done [15] and to perform this we must elect the listed techniques: tension band, screw fixation, cerclage wiring, partial and total patellectomy [13, 14].

Excessive comminution, impossibility in fracture reduction, surgical fixation failure and chronic infection led to a total patellectomy, being considered a salvation procedure [5]. The polar fractures chirurgic treatment can be divided regarding the presence of comminution. When it is present only in one of two poles and there is no possibility of reduction, the partial patellectomy is indicated, but the greatest fragments must be maintained to preserve the benefit of the extensor mechanism which could be close to normal [8, 16]. When the polar fracture, base or apical, was not comminuted, several treatments may be employed such as suture, tension band, cerclage wire, screw fixation or the association of these techniques [5]. For transverse patella fractures, the modified tension band in most times is the better choice. We choose to use this traditional and well-established technique which consists in fixate the fracture with tension band and two parallel K-wires [11, 17]. The tension band was not used in circular form which was originally described, but it was employed and shaped as an anterior eight as described by Cramer and Moed [11]. Considering the fracture pattern, a vertical patella fracture, we conduct a technique modification and applied K-wires orthogonal to the fracture line (Fig. 4-6), and this enabled that the tension forces in the extensor mechanism of the knee were transformed to compression forces when the joint was flexed [17, 18], resulting in biomechanical stimulation for fracture consolidation.

The Gustilo and Anderson [10] classification in this case a type IIIA is shown in literature with very low incidence. Regarding the Gustilo and Anderson [10] classification, Torchia and Lewallen [9] described in their study about open patella fracture that 14.5% of all open patella fracture were classified as type I, 76.4% were type II, 1.8% were type IIIA and 7.3% were type IIIB. Catalano et al [7] and Pailo et al [6] reported similar incidences of open fracture in this joint, 15% type I, 53% type II and 32% type III. At present, no article was found reporting the incidence of open vertical patella fracture. To the best of our knowledge, this is an atypical presentation of patella fracture which includes two conditions that were reported separately in the literature with low incidences.

Immediate fixation as performed in this case is recommended in type I, II and IIIA [9, 19, 20] without any increase in infection rates, joint functionality change [9, 21-23] and this premature stabilization benefits infection prevention due to the neovascularization and the increased tissues perfusion [20]. Additionally there is no evidence that the metal itself increases the infection rates [24].

Open patella fractures present higher incidences of complications as compared with closed fractures, and the most frequent are infection and non-union with respective rates of 0-5% and 0-3% to closed fractures [8, 16], and 10.7% and 7.1% to open fractures [9, 25].

The patient’s clinical and functional final evaluation was similar to other works such as Pailo et al [6] that showed 53.3% of good results are considered to be those in which the patient did not have pain, joint swelling and had a range of motion at least 110º of flexion and full extension. Torchia and Lewallen [9] also demonstrated similar outcomes. Final functional result is not determinate by an isolated factor, but rather by the association of several factors as the mechanism of trauma, presence of associated fractures, patient age, pattern fracture and the employed treatment [6]. It deserved to highlight the early mobilization of joint that showed better results in the final range of motion [6] and in the nutrition of articular cartilage [4], as opposed to late mobilization which determined incontestably a stiffness more sharply knee [6].

Small functional limitation presented by patient is justified by the high-energy mechanism of trauma and exposure of the fractured cartilage, even so the patient had a good functional joint which demonstrated that the early chirurgic treatment applied and the precocious articular mobilization improve prognosis, suggesting that the employment of these practices should be adopted whenever possible. We also have to consider that the fracture was not comminuted and the young age of the patient which positively influenced the final outcome.
